# Evaluation of one-shot vincristine sulfate combined with surgical excision as a new regimen for treatment of canine transmissible venereal tumor

**DOI:** 10.5455/javar.2024.k805

**Published:** 2024-08-26

**Authors:** Khaled Abouelnasr, Mohamed A. Hamed, Rasha Eltaysh, Eman Abo Elfadl, Shefaa Bazeed, Samah Ibrahim, Liana Fericean, Foad Farrag, Mohamed Salem, Awad Rizk

**Affiliations:** 1 Department of Surgery, Anesthesiology and Radiology, Faculty of Veterinary Medicine, Mansoura University, Mansoura, Egypt; 2 Department of Surgery, Anesthesiology and Radiology, Faculty of Veterinary Medicine, Aswan University, Aswan, Egypt; 3 Department of Pharmacology, Faculty of Veterinary Medicine, Mansoura University, Mansoura, Egypt; 4 Department of Husbandry and Development of Animal Wealth, Faculty of Veterinary Medicine, Mansoura University, Mansoura, Egypt; 5 Department of Biochemistry and Chemistry of Nutrition, Faculty of Veterinary Medicine, Badr University, Badr, Egypt; 6 Department of Clinical Sciences, College of Medicine, Princess Nourah bint Abdulrahman University, Riyadh, Saudi Arabia; 7 Department of Biology and Plant Protection, Faculty of Agriculture. University of Life Sciences, Timișoara, Romania; 8 Department of Anatomy and Embryology, Faculty of Veterinary Medicine, Kafrelsheikh University, Kafrelsheikh, Egypt; 9 Department of Basic Veterinary Sciences, Faculty of Veterinary Medicine, Delta University for Science and Technology, Dakahlia, Egypt; 10 Department of Veterinary Clinical Sciences, Faculty of Veterinary Medicine, Jordan University of Science and Technology, Irbid, Jordan

**Keywords:** Transmissible venereal tumors, surgical excision, dogs, vincristinesulphate

## Abstract

**Objective:**

The purpose of this study was to investigate the effectiveness of surgical excision combined with a single shot of vincristine sulfate for treating transmissible venereal tumor (TVT) in dogs.

**Materials and Methods:**

Fifty-two dogs were divided randomly into two groups (*n = *26). Dogs in Group I were treated surgically by debulking the tumorous mass, whereas dogs in Group II were subjected to a combination of surgery and a single injection of vincristine sulfate.

**Results:**

Female dogs showed a high prevalence of TVT compared to males (67.3%, *n = *35 *vs.* 32.7%, *n = *17), respectively. The German shepherd’s breed showed a high prevalence of TVT compared to other breeds. There was a positive association between sex and outcomes. Most occurrences of regret in Group II were reported in females (*n = *16, 61.5%) compared to male dogs (*n = *10, 38.5%). There was also a positive association between breed and outcome. Most occurrences of regret in Group II were reported in German shepherd dogs (*n = *16) compared to Group I (*n = *7). In Group I, 15 dogs (57.7%) showed a complete regression, and 11 (42.3%) underwent recurrence. However, in Group II, 21 dogs (80.7%) showed a complete regression, and 5 dogs (19.2 %) underwent recurrence.

**Conclusions:**

It appears therefore that the simultaneous use of surgery and administration of a single dose of vincristine sulfate could be considered a combination therapy for TVT as it reduces the risk of recurrence and has a reasonable cost. Recognizing potential risk factors associated with TVT in dogs may be helpful in constructing the best preventive measures.

## Introduction

Transmissible venereal tumor (TVT) is a horizontally transferred venereal round cell tumor identified in canines [1,2]. It is a naturally occurring tumor that mostly affects the external genitalia and occasionally the interior genitalia; however, in certain circumstances, it can also be seen in extra-genital regions. Live tumor cells are passed from one animal to another through sexual contact, leading to the spread of the disease [3]. The tumor cell itself is assumed to be the transmissible factor causing this sickness [4]. The major histocompatibility complex prevents the transmission of this infectious malignancy between dogs and among the members of the Canidae family, which includes foxes, coyotes, wolves, and jackals [5].

TVT typically affects the external genitalia of both sexes of dogs. Males often have the tumor in the glans, the caudal portion of the penis, or the foreskin. TVT frequently develops in females at the intersection of the vestibule and the posterior portion of the vagina, and it sometimes develops at the urethral orifice [6]. This illness causes discomfort, hemorrhagic, and serosanguineous discharge from the external genitalia in dogs. TVT is usually friable, red to flesh in color, and cauliflower-like in appearance. Unless the tumor obstructs the urethral opening, becomes necrotic and infected, or has metastasized, the overall health of the afflicted dogs is unaffected [7].

The plan for treatment with the best chance of success is the total surgical excision of localized malignancies without metastatic involvement. In dogs with stage I illness, tiny, easily accessible, well-differentiated carcinomas, and non-invasive tumors, surgical removal may be effective. Another reason for recurrence is the invasion of the surgical area by TVT cells [8]. A complete surgical excision combined with vincristine sulfate (0.5 mg/m^2^) IV chemotherapy administered once per week for 3–6 weeks is successful [9]. Due to the anatomic placement of many of these tumors, total surgical excision is frequently not possible. If no more radiation or chemotherapy is administered, recurrence is probable [10].

Chemotherapy has been shown to be the most effective and practical form of treatment. Chemotherapy has several benefits, including a high percentage of cure, simplicity in administration, and a possible benefit for multi-focal metastatic cancer. Chemotherapy drugs used to treat TVT involve antimitotic agents such as cyclophosphamide, methotrexate, vincristine, vinblastine, or doxorubicin, with vincristine sulfate being the most commonly utilized medication. [11,12]. The most efficient, suitable, and safe chemotherapy drug is vincristine when used as a single agent, curing even individuals with extra genital metastasis [13]. In instances treated in the early phases of development, notably in cases lasting less than a year, and regardless of whether metastasis is present or not, a cure rate of close to 100% is attained. Longer therapy sessions are needed, and the cure rate is lower in instances with longer lengths [14]. Less than 15% of the dogs that received vincristine treatment had adverse effects from the medication [15].

To the best of the authors’ understanding, there is a low amount of data regarding the risk factors associated with TVT in dogs. It was hypothesized that the results of the current experiment could be in close agreement with the previous studies (combination therapy of surgery plus administration of vincristine sulfate) as an effective and efficient treatment for TVT in dogs. Therefore, the objective of the current research was to explore the effectiveness of the simultaneous use of surgical excision and a single shot of vincristine in the treatment of TVT in dogs and to highlight the prevalence of TVT in dogs in Egypt.

## Materials and Methods

### Ethics approval

The Mansoura University’s Animal Care Committee (MU-ACUC) approved this study following Egyptian ethical guidelines for research involving laboratory animals (VM. R.22.10.15 R1). All procedures were followed in compliance with the applicable norms and legislation. The research was done in accordance with the ARRIVE (Animal Research: Reporting of *In Vivo* Experiments) criteria.

### Dogs

A total of 52 dogs with a mean body weight (BW) of 27.76 ± 16.48 kg, were brought to Mansoura Veterinary Teaching Hospital, Mansoura University, Mansoura, Egypt, with a finding of serosanguineous discharge and granulomatous (tumorous) lesions found in and around their genitalia. Histopathology and pathognomonic findings supported the diagnosis of the disease [9].

Animal risk factors such as age, sex, breed, season, pet care, neuter status, genital and extragenital lesions, bleeding, and inflammation were gathered from the owners’ anamnesis of their pets. These criteria were recorded and assessed by a solitary person as clinical index scores to be quantified and analyzed in an unbiased way (Table 1). Table 1 is the clinical index score for the clinical findings and treatment outcome of TVT in dogs, which was modified from [16].

### Treatment protocols

Before surgery, feed was withheld for 4–6 h, and patients were given a pre-operative dose of broad-spectrum antibiotics amoxicillin and flucloxacillin. (Flumox, E.I.P.I.C.O, Cairo, Egypt). Every dog received premedication by injecting atropine sulfate at a dose of 0.1 mg/kg (Atropine 1 mg/ml, Adwia, Cairo, Egypt), followed by injecting xylazine HCl at a dose of 1 mg/kg (Xylaject, Adwia, Egypt). Thiopental sodium 2.5% (Thiopental sodium, E.I.P.I.C.O, Egypt) was used to induce and sustain general anesthesia through intravenous administration (IV).

**Figure 1.**
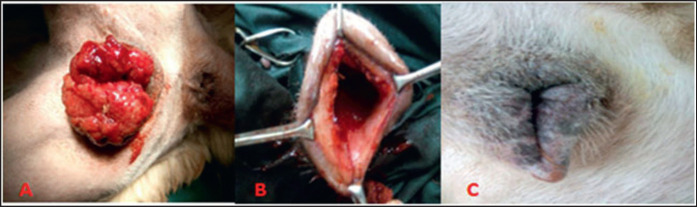
TVT in a female dog. A: cauliflower-like granulomatous mass protruding out of the vulva; B: Complete regression after surgery and one shot of vincristine chemothearpy; C: after 6 months of follow-up.

The site of the operation was aseptically prepared. Dogs in group I were treated surgically, in which the tumorous mass was excised [15]. While dogs in group II received a combined treatment of surgery and chemotherapy (Fig. 1). Following the surgical debulking of the tumorous mass, a single shot of vincristine sulfate (2 mg/2 ml; Oncovin, Lilly, France) was administered through gradual IV injection as a 0.01% solution at a dose of 0.025 mg/kg BW [17].

### Post-operative care and follow-up 

The evaluation of the treated dogs was based on how quickly the wound was healed after surgical excision and recurrence during the first 6 months after treatment. Unfavorable side effects of surgery and chemotherapy were also noted. To determine the recurrence rate, follow-up data (6 months) was gathered through phone contact with the owners.

### Histopathological examination

Each dog had a 1 × 1 cm^2^ biopsy taken from granulomatous lesions. Biopsies were then stored in 10% neutral buffered formalin. Each specimen was then prepared for histological analysis to check for possible changes in the tissue and cellular structures [18].

### Statistical analyses

The computer application SPSS (Statistical Package for Social Science), version 23.0, was used to tabulate, code, and subsequently analyze the data. The distribution of frequencies in TVT was determined using Fisher’s exact tests. The correlation between factors for a Spearman was determined using crosstabs. For all analyses, a difference was considered significant if the *p-*value was < 0.05. To investigate the relationship between additional risk variables and the TVT result, a logistic regression analysis was performed. Additionally, univariate logistic regression statistics were run. In a multivariable test, the outcome showed that the model’s estimates matched the data at a respectable level. *p-*value, odds ratio (OR), and confidence interval (CI: 95%) were the parameters included in the findings for each variable. The findings of each statistical analysis were deemed significant at *p *< 0.05.

**Table 1.** Clinical index score for the clinical findings and treatment outcome of TVT of dogs, modified according to [16].

Parameters	Score and description
Age (Year)	0 = Less than one year 1 = 1-5 2 = > 5
Sex	0 = Male 1 = Female
Breed	0 = German 1 = Baladi 2 = Others
Pet care	0 = Poor 1 = Adequate 2 = Good
Neuter status	0 = Spayed or castrated 1 = Intact
Genital lesion	0 = Mass 1 = Nodule
Extragenital lesion	0 = Present 1 = Absent
Bleeding	0 = Yes 1 = No
Inflammation	0 = Present 1 = Absent
Interference	0 = Surgical 1 = Surgical plus chemotherapy
Outcome	0 = No recurrence 1 = Recurrence
Regression	0 = No regression 1 = Total regression

## Results

**Figure 2.**
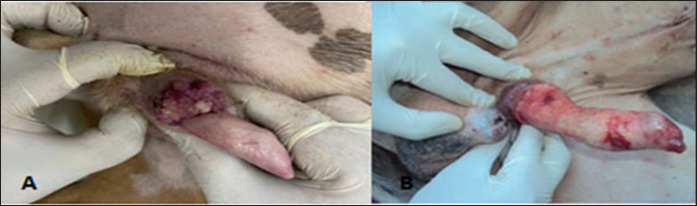
TVT in the shaft of the penis of a male dog. A: cauliflower-like granulomatous mass at the shaft of the penis; B: Complete regression after surgery and one shot of vincristine chemotherapy.

In the present study, the animal-level risk factors associated with TVT in dogs were estimated. Female dogs showed a high prevalence of TVT compared to males (67.3%, *n = *35 vs. 32.7%, *n = *17, *p < *0.05), respectively (Fig. 2) and (Table 2). TVT was more prevalent in ages 1–5 years (*n = *35, 67.3%), compared to both ages more than 5 years (*n = *13, 25.0%), and ages less than one year (*n = *4, 7.7%) (*p *< 0.05). The German shepherd breed was the most prevalent dog breed in the present study affected by TVT compared to other breeds (26 *vs.* 10, *p < *0.01). Caring of the dogs played a significant role (*p *< 0.05) in the prevalence of TVT, as poor caring of pets increased the incidence of TVT (*n = *32, 61.5%) compared to good caring (*n = *6, 11.5%). However, the incidence of TVT was higher in intact dogs than in others (42 *vs.* 10, *p *< 0.05). Genital bleeding (*n = *30, 57.7%) and secondary inflammation (*n = *32, 61.5%) as TVT lesions were primary features of TVT in both genders. Eight of the TVT cases had extra genital TVT lesions, with three in the nose and five in the skin. Genital lesions in the form of mass were higher than in nodular form (73.1%, *n = 38 vs.* 26.9%, *n = *14, *p < *0.05).

**Table 2.** The relation of potential risk factors with prevalence of TVT in dogs. *p*-value < 0.05 was deemed significant.

	Risk factor	Number	Prevalence (%)	*p*-value	df
Age	Less than 1 year	4	7.7	0.00	2
	1–5	35	67.3		
	> 5	13	25.0		
Sex	Male	17	32.7	0.006	1
	Female	35	67.3		
Breeds	German	26	50	0.001	2
	Baladi	16	30.8		
	Others	10	19.2		
Pet care	Poor	32	61.5	0.00	2
	Adequate	14	26.9		
	Good	6	11.5		
Neuter status	Spayed/castrated	10	19.2	0.001	1
	Intact	42	80.8		
Genital lesion	Mass	38	73.1	0.001	1
	Nodule	14	26.9		
Extragenitallesion	Present	8	15.4	0.00	1
	Absent	44	84.6		
Bleeding	Yes	30	57.7	0.267	1
	No	22	42.3		
Inflammation	Present	32	61.5	0.096	1
	Absent	20	38.5		

In Group I, complete surgical excision of tumor mass was performed in 26 dogs (50%). The dogs have been checked again at 8–week intervals during 6 months; 15 dogs (57.7%) had a complete regression, and 11 dogs (42.3%) underwent recurrence. In Group II, a combination of complete surgical excision of the tumor mass with a single shot of vincristine was performed in 26 dogs (50%); 6 months later, 21 dogs (80.7%) had complete regression and 5 dogs (19.2%) underwent recurrence.

There was a positive association between sex and outcome (*p *< 0.05; OR: 2.3; CI 95%: 5.1–1.07). Most occurrences of regret in Group II were reported by females (*n = *16, 61.5%) as opposed to male dogs (*n = *5, 19.2%). In Group I, there were more females (*n = *12) than males (*n = *3). There was also a positive association between breed and outcome (*p *< 0.05; OR: 2.30; CI 95%: 1.09–4.81). Compared to other breeds, most occurrences of regret were reported in German shepherd dogs in Group II (*n = *16) and Group I (*n = *7).

There were non-significant correlations between age, pet care, neutral status, genital lesion, extral gemnita lesion, bleeding, and inflammation with TVT outcome in dogs (*p* = 0.460, *p =* 0.012, *p =* 0.701*, p =* 0.201, *p =* 0.463, *p =* 0.995, *p =* 0.982, respectively).

**Figure 3.**
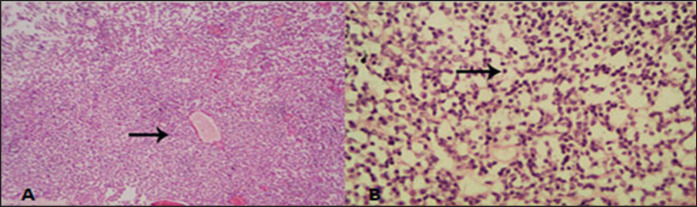
A; Small round cells in radial arrangement around blood vessels with few fibrovascular stroma (HE 10x). B; Small cell with hyperchromasia and little pleomorphism (HE, 40x).

On histopathological examination, hematoxylin-eosin-stained biopsy samples revealed small round cells in a radial arrangement around blood vessels with little fibrovascular stroma (Fig. 3A). A small cell with hyperchromasia and little pleomorphism was also seen (Fig. 3B).

## Discussion

Currently, TVT is one of the most common conditions affecting the external genitalia of dogs [16,19]. This is alluding to one of the common causes of surgical intervention in dogs. The objective of the present research was to examine the efficacy of surgical excision in conjunction with a single vincristine injection for the treatment of canine TVT, with a particular focus on the prevalence of TVT in Egyptian dogs, due to the paucity of literature regarding the condition’s incidence.

Compared to other ages, TVT was more common in the ages of 1–5 years. This finding agrees with those of [16,20], who also noted that TVT was more common in young than adult middle-aged dogs, mainly because adult dogs are more sexually active at these ages and are hence more vulnerable to exposure.

The current study showed that there are variations in the occurrence of TVT among different dog breeds, with German shepherds having the highest prevalence compared to other breeds analyzed. The reason for this may be the large population of German Shepherds in Egypt, as they are known for their excellent guarding abilities [21].

Female dogs were the gender most frequently impacted by TVT in the recent study compared to male dogs. Unlike female dogs, who go into heat only once every 6–7 months, male dogs are always susceptible and therefore have a higher risk of spreading the disease. It was shown that a single TVT-affected male dog disseminated the disease to 11 out of 12 female dogs, and at least in some places, females are inherently more likely to contract the disease than males are. As a result, females were shown to have a higher incidence of venereal granuloma than males [22].

Extragenital TVT lesions were pronounced in eight dogs. This might be attributed to specific actions such as sniffing, licking, and biting, which are crucial in the spread of tumor cells to the nose, mouth, and skin from the initial site. TVT can spread to multiple internal organs through lymphatic or hematogenous pathways and may be more likely to happen in animals with weakened immune systems [23–25].

The quality of pet care, neuter status, or TVT status did not appear to be significantly correlated. This could be a result of misinterpretation or a small sample size. For instance, one study substituted stray status for inadequate pet care. It is recommended that more research be done to ascertain whether TVT and issues of dog management in Egypt are related [16].

Genital growth and bleeding were shown to be prevalent TVT signs in the current investigation, and may therefore be more helpful in the clinical diagnosis of TVT in dogs. This result is similar to that of the previous study [26]. The eventual development of genital growth in dogs with TVT was significantly influenced by sex differences, with male dogs being more likely to develop genital growth [16]. TVT most often affected the prepuce and caudal aspects of the penis were the most often affected areas by TVT, making genital masses visible. The tumor’s location in these regions reveals its mode of dissemination. However, the deep position of these growths in the vestibule-vagina junction or vagina in female dogs may account for the absence of genital visible growths in these animals. TVT is frequently associated with bloody preputial or vaginal discharge, which may make it easy to diagnose clinically in female dogs. Secondary inflammation, manifesting as lymphocyte infiltration of the diseased lesion, may cause a low degree of lymphopenia [25].

Traditionally, surgery is the most widely used method in the treatment of TVT; however, inaccessibility of the site, incomplete excision, and metastasis may explain the recurrence of TVT following surgery [15,27]. In the current study, out of the 26 dogs treated surgically (Group-I), 11 underwent incomplete regression, resulting in a 40% recurrence rate. The recurrence could be due to a weak lymphoblastogenic response [25]. Additionally, the tumor mass could not be completely excised in 2 dogs where the lesion was inaccessible due to its spread to the surrounding tissues. Even a complete episiotomy incision could not help. Of these 2 cases, at the end of the 6-month study period, one had a completely regressed lesion, whereas the other had a regeneration. This post-operative self-regression may be due to the provocation of the body’s own immune system with the release of tumor cells in the circulation and the ensuing lymphoblastogenesis, as well as the humoral response.

Vincristine sulfate, an alkaloid derived from Vinca rosa, has also been utilized as a common anti-cancer medication in animal medicine due to its mild adverse effects. It is an antimitotic agent that inhibits mitosis by stopping spindle production [28]. Following the injections of vincristine sulfate at a dosage of 0.025 mg/kg bw, a percentage of full regression was (80.33%) in dogs [29]. Vomiting and inappetence were observed in one dog during the first 48 h. In this study, a single injection of vincristine sulfate after surgery to remove a tumor mass caused 80.7% regression. However, 5 dogs (19.2%) had recurrence as described [29]. Using vincristine sulfate may not only help the dogs get better faster, but it may also help lessen some of the bad effects of the cancer treatment, like severe leukopenia and thrombocytopenia [13,30].

Histological examination in the present research revealed cells organized in a radial formation surrounding blood vessels, with minimal fibrovascular stroma. Furthermore, we observed minimal pleomorphism and hyperchromasia. These results, which served as the foundation for the confirmed diagnosis of TVT in the current investigation, are consistent with the explanation provided [9].

The presentation of the study’s limitations is necessary. Initially, the limited number of dogs studied could potentially impede the ability to draw definitive conclusions. Furthermore, not all excised TVT masses received histopathological confirmation. Third, using only vincristine sulfate hinders the evaluation due to the absence of a chemical group. Traditional methods of gathering data and innovative diagnostic techniques can help address these constraints.

## Conclusion

In conclusion, as a result, we advise utilizing surgery together with a single dose of vincristine sulfate as a combination therapy to reduce the risk of recurrence at a reasonable cost for TVT in dogs. Recognizing the potential risk factors associated with TVT in dogs may help construct the best preventive measures. Additional research is necessary to confirm the laboratory results of treated canines.

## List of Abbreviations

CI: 95%, Confidence interval; IV, Intravenous; OR, odds ratio; TVT, transmissible venereal tumor
